# The Impact of Psychosocial Factors on Pain and Functional Outcomes After Rotator Cuff Repair: A Systematic Review

**DOI:** 10.2174/0117450179479980260803105712

**Published:** 2026-08-05

**Authors:** Theodoros Paschalis, Monique Gilmore, Myrella Paschali, David Endell, Markus Scheibel, Asimina Lazaridou

**Affiliations:** 1 Department of Psychiatry, Cambridgeshire and Peterborough NHS Foundation Trust, Cambridge, UK; 2 Department of Anesthesiology, Perioperative and Pain Medicine, Brigham & Women’s Hospital and Harvard Medical School, Boston, Massachusetts, USA; 3 Klinik und Poliklinik für Psychiatrie und Psychotherapie, Heinrich-Heine-Universität Düsseldorf, Düsseldorf, Germany; 4 Department for Shoulder and Elbow Surgery, Schulthess Clinic, Zürich, Switzerland; 5 Center for Musculoskeletal Surgery, Charité-Universitaetsmedizin, Berlin, Germany

**Keywords:** Rotator cuff repair, Postoperative pain, Psychological factors, Depression, Anxiety, Pain catastrophizing, Resilience, Personalized medicine, Outcome prediction

## Abstract

**Introduction:**

Outcomes after rotator cuff repair are commonly evaluated in terms of structural healing and surgical technique. However, psychological factors may also influence patients’ experience of pain and functional recovery. Increasing attention has therefore been directed toward the potential role of preoperative psychological distress in shaping postoperative outcomes.

In this review we aimed to systematically review the available evidence examining whether psychological factors present before surgery are associated with postoperative pain and functional outcomes following rotator cuff repair.

**Methods:**

This systematic review was conducted in accordance with PRISMA guidelines and prospectively registered in PROSPERO (CRD420251132190; 28/08/2025). A structured literature review was performed using MEDLINE, Embase and PsycINFO, from database inception to March 6, 2026, for studies evaluating preoperative psychological variables in relation to postoperative pain and functional outcomes. Eligible study designs included randomized controlled trials, non-randomized trials, and observational cohort studies; however, only observational cohort studies met the final inclusion criteria. Risk of bias was assessed using the ROBINS-I tool, and methodological quality was appraised using the Newcastle-Ottawa Scale.

**Results:**

Ten observational studies were included. Across these studies, higher levels of depression, anxiety, pain catastrophizing, poorer emotional well-being, negative affect, and broader psychological distress were frequently associated with worse postoperative pain intensity and lower functional outcome scores. The reported associations were generally small-to-moderate strength and varied across cohorts. Some findings suggested potential interaction between psychological vulnerability and biological predisposition, including variation in Catechol-O-Methyltransferase (COMT) genotype. Importantly, elevated preoperative psychological distress did not preclude meaningful postoperative improvement, and several studies reported parallel improvements in psychological symptoms and physical recovery over time.

**Discussion:**

The available evidence indicates that recovery after rotator cuff repair cannot be explained solely by structural or surgical factors. Psychological health appears to influence how patients experience and report postoperative pain and function. However, the observed relationships are not uniform, and the evidence base is limited by observational design, methodological heterogeneity, and variability in outcome measurement. While preoperative psychological assessment may have clinical value, further prospective and interventional research is required to clarify its impact on surgical outcomes.

**Conclusions:**

Preoperative psychological distress is associated with postoperative pain and functional outcomes after rotator cuff repair, although it does not determine recovery in isolation. Psychological factors likely represent one component of a broader multifactorial recovery process. Future studies should explore whether targeted psychological interventions can improve postoperative pain trajectories and functional recovery.

## 1 INTRODUCTION

Rotator cuff repair outcomes have traditionally been considered in terms of structural healing and surgical technique. Despite the importance of rotator cuff and soft tissue function in shoulder biomechanics, relatively few studies have systematically evaluated their influence on postoperative recovery trajectories after shoulder arthroplasty [1]. Increasingly, however, attention has shifted toward patient-related factors that may influence recovery. In both clinical practice and research, postoperative outcomes are commonly assessed using measures of pain intensity alongside patient-reported functional scores. Psychological health may therefore play an important role in shaping the recovery experience after surgery.

### 1.1 Previous Systematic Reviews

Several systematic reviews have examined the role of psychosocial factors in recovery after rotator cuff surgery. Panattoni *et al.* [2] reported associations between psychological distress and poorer pain and functional outcomes, although study heterogeneity limited firm conclusions. Kennedy *et al.* [3] also identified depression, anxiety and patient expectations as relevant to postoperative recovery, even though most patients experienced pain relief after surgery. In a broader review of musculoskeletal procedures, Coronado *et al.* [4] described weak to moderate relationships between psychological variables and postoperative pain, function and disability. The authors noted the limited number of high-quality studies available at the time. A review of 39 studies reported that depression, anxiety, distress and sleep disturbance were common among patients with rotator cuff tears and were associated with greater pain and reduced function [5].

### 1.2 Depression and Anxiety

Depression and anxiety are the most widely examined psychosocial factors in patients undergoing rotator cuff repair. Patients reporting higher levels of these symptoms before surgery tend to have poorer pain and functional scores at baseline, and this pattern often persists after the procedure [6]. Longo *et al.* [7] also observed negative correlations between anxiety, depressive symptoms, and functional recovery. Despite overall improvement after surgery, individuals with greater psychological distress typically start from a lower functional level and continue to report comparatively higher pain postoperatively. Cho and colleagues [8] similarly associated depressive and anxious symptoms with increased pain and disability, while their subsequent work suggested that rotator cuff repair may be accompanied by improvements in psychological wellbeing and quality of life [9]. Evidence from more recent cohorts indicates that established diagnoses of depression and anxiety are linked with less favourable postoperative pain and functional outcomes [10]. At the same time, depressive symptoms themselves may lessen after repair, even where pain remains elevated relative to patients without depression [11]. This pattern supports a reciprocal relationship between psychological state and recovery.

### 1.3 Overall Mental Health

Emotional well-being and distress add another dimension to recovery outcomes. Poorer emotional health was found to be linked with greater pain and disability, mediated by a worse perception of shoulder status [12]. This was supported by findings from Potter *et al.* [13] who noted that distress undermined patients’ own assessment of shoulder function. Additionally, Şahinoğlu *et al.* [14] confirmed associations between low emotional health and higher pain, disability, and impaired quality of life. Contrasting this, other work showed that mild to moderate distress was not strongly related to recovery, suggesting that only more severe psychological symptoms exert a significant influence [15]. Psychological burden also affects patients’ experience before surgery. Both Okafor *et al.* [16] and Gibson *et al.* [17] demonstrated that individuals with greater distress reported more severe preoperative pain. Nevertheless, in shoulder arthroplasty, psychological distress should not be viewed solely as a preoperative predictor, but rather as part of a complex and potentially bidirectional interaction between chronic pain, disability, and recovery [18].

### 1.4 Preoperative Psychosocial Factors, Genetics and Pain

Gibson *et al.* [17] identified a moderate correlation between catastrophising and reported disease severity. In chronic pain populations, pain catastrophizing appears to interact with Catechol-O-Methyltransferase (COMT) genotype, and this interaction has been linked to greater daily pain, suggesting a gene-cognition influence on pain modulation [19]. Catastrophizing involves magnification of pain-related stimuli, feelings of helplessness, and persistent rumination. In shoulder pathology, greater catastrophising has been linked with higher preoperative pain and, among individuals with a low COMT diplotype, poorer postoperative outcomes [20]. Patients classified as high risk based on catastrophising and COMT genotype have also reported greater fear and movement-evoked pain at 12 months, with depressive symptoms mediating part of this relationship [21]. These findings align with broader pain research, where catastrophizing is regarded as an important contributor to pain perception [22, 23]. The biopsychosocial model describes recovery as influenced by biological, psychological, and social factors [24]. Spotting psychological vulnerability before surgery could therefore support more tailored management strategies.

### 1.5 Systematic Review Aim

This systematic review aims to synthesize current evidence on whether preoperative psychological factors influence postoperative pain intensity and functional recovery after rotator cuff repair. Addressing this question may have important implications for orthopaedic clinical practice. Should psychological factors strongly influence recovery, routine screening and targeted interventions could improve outcomes and help reduce opioid reliance postoperatively and longer term. Surgeons increasingly face the challenge of balancing effective pain control with the risks of prolonged opioid use, and evidence suggests that patients with higher psychological distress are more likely to seek additional medication [25]. Psychological factors examined in this review are considered within a broader psychosocial framework that recognises the interaction between emotional, behavioral, and contextual influences on postoperative recovery.

## 2 METHODS

### 2.1 Data Sources, Search Strategy and Study Selection

This review followed the Preferred Reporting Items for Systematic Reviews and Meta-Analyses (PRISMA) guidelines. The methodological approach was discussed and agreed upon by the study team prior to initiation. The protocol was prospectively registered in the PROSPERO database (registration number: CRD420251132190; registration date: 28/08/2025).

We searched MEDLINE, Embase, and the PsychINFO in duplicate (TP, MG) for studies evaluating pre-operative psychological status prior to rotator cuff repair surgery and postoperative recovery and pain patterns, from inception to March 6, 2026 (full search strategy shown in **Appendix 1**). The search strategy combined terms related to the operative procedure (“rotator cuff surgery,” “rotator cuff repair”) with terms relating to pre-operative states (“depression”, “anxiety”, “catastrophising”, “kinesiophobia”) and post-operative outcomes (“pain”). Titles and abstracts were screened independently, followed by full-text review of eligible studies, again in duplicate (TP, MG). Reference lists of included articles were also reviewed to identify additional relevant studies. Grey literature sources were not included, as the review focused on peer-reviewed studies to ensure methodological rigor and consistency in study quality. Any discrepancies were resolved by a third investigator (MP). While formal inter-rater reliability statistics (*e.g.*, Cohen’s kappa) were not calculated, this method is consistent with established systematic review methodology. The study selection process is summarized in the PRISMA flow diagram (Fig. **1**), and full search strategies for each database are provided in **Supplementary Material 1** [35].

### 2.2 Inclusion and Exclusion Criteria

Eligible studies investigated the association between preoperative psychosocial factors and postoperative pain and functional outcomes after rotator cuff surgical repair. Randomized controlled trials, non-randomized trials, and observational designs were eligible for inclusion; however, no randomized or non-randomized trials met the inclusion criteria. No restrictions were placed on geographic location or year of publication. Included studies were required to report a quantitative statistical association (*e.g*., correlation coefficient, regression estimate, mediation analysis, or predictive modelling) between preoperative psychological variables and postoperative pain or functional outcomes. Studies were excluded if they were incomplete, non-human, qualitative, single case reports or opinion pieces, or if they investigated only preoperative pain or postoperative psychological factors. We included studies that examined associations between psychological factors and clinically relevant outcomes, even where these were not reported as correlation coefficients, provided that the statistical analysis demonstrated a clear relationship between variables (*e.g.*, regression analyses or group comparisons). This approach was adopted to ensure that clinically meaningful evidence was not excluded solely on the basis of reporting format.

### 2.3 Data Extraction

Two authors (AL, MP) independently extracted data using a pilot-tested form. Extracted information included: study author, year of publication, journal, sample size, psychological factors assessed, measurement instruments, pain outcomes, statistical analyses, demographic variables, and main findings.

### 2.4 Assessment of Methodological Quality

Two authors (AL, TP) independently assessed methodological quality. The ROBINS-I tool (Risk of Bias in Non-randomized Studies of Interventions) was used for non-randomized and observational studies. Study quality was additionally appraised with the Newcastle-Ottawa Scale (NOS). Discrepancies were resolved by consensus, with a third reviewer (MP) consulted when required.

## 3 RESULTS

Ten studies met the inclusion criteria, all of which were observational cohort studies, including prospective and retrospective cohort designs (Table **1**). ROBINS-I was used to assess risk of bias in included non-randomized cohort studies (Table **2**), and all studies were also evaluated using the NOS for methodological quality (Table **3**). While evaluating these studies with these 2 tools, it was noted that in some instances studies received high quality scores on NOS despite moderate risk of bias scores on ROBINS-I [15]. These discrepancies may be explained by the fact that NOS assesses broader study quality and awards points for general characteristics such as the use of a validated measurement tool (which was the case in the included studies). In contrast, ROBINS-I more strictly assesses for bias by comparing to the gold standard of randomized-controlled trials and easily penalizes observational studies for baseline confounding.

Each study investigated the relationship between preoperative psychological factors such as depression, anxiety, pain catastrophizing, emotional well-being, psychological resilience, and general psychological distress and postoperative pain and functional outcomes following rotator cuff repair surgery. A range of validated instruments were used to assess psychological status preoperatively (*e.g*., Pain Catastrophizing Scale [PCS], Hospital Anxiety and Depression Scale [HADS], 36-Item Short Form Survey Mental Component Score [SF-36 MCS], Patient Health Questionnaire-9 [PHQ-9], Patient-Reported Outcomes Measurement Information System [PROMIS], Brief Resilience Scale [BRS], Distress and Risk Assessment Method [DRAM], Depression Anxiety Stress Scale [DASS], Pain Self-Efficacy Questionnaire [PSEQ], Tampa Scale of Kinesiophobia-11 [TSK-11], and the anxiety/depression dimension of the EuroQol 5-Dimensions 5-Levels questionnaire [EQ-5D-5L]) and postoperative outcomes (*e.g*., Visual Analog Scale [VAS], American Shoulder and Elbow Surgeons score [ASES], Shoulder Pain and Disability Index [SPADI], Western Ontario Rotator Cuff Index [WORC], Quick Disabilities of the Arm, Shoulder and Hand [QuickDASH], 12-Item Short Form Health Survey [SF-12], Simple Shoulder Test [SST], University of California Los Angeles shoulder score [UCLA], Numeric Rating Scale [NRS], and PROMIS Pain Interference scores). A summary of the results from the studies included, as well as the level of evidence strength, is shown in Table **1**.

### 3.1 Pain Catastrophizing and Depression

Simon *et al.* [21] examined the relationship between pain catastrophizing, depressive symptoms, and postoperative outcomes. Depressive symptoms were assessed using the PHQ-9, and catastrophizing was measured with the PCS. Higher preoperative levels of both variables were associated with worse postoperative pain interference on the PROMIS scale. Higher pain catastrophizing was moderately associated with greater postoperative pain interference, and depressive symptoms were similarly associated with worse postoperative pain interference. These associations persisted after adjustment for baseline pain and function. The study also considered the role of genetic variation. Individuals with a high pain-sensitivity COMT genotype who reported elevated PCS scores experienced greater postoperative pain than other groups. This pattern points to an interaction between psychological vulnerability and genetic susceptibility in influencing postoperative pain.

In addition, Cho *et al.* [9] evaluated preoperative depression and anxiety using the HADS in patients undergoing arthroscopic rotator cuff repair. Although psychological symptoms and quality-of-life measures improved following surgery, baseline depression and anxiety scores were not significantly associated with postoperative pain intensity or functional outcomes at 12-month follow-up. These findings suggest that while depressive symptoms may improve alongside clinical recovery, their prognostic value for postoperative pain outcomes may be variable across patient cohorts.

### 3.2 General Psychological Distress and Functional Recovery

Lau *et al.* [6] explored how both general psychological distress and shoulder-specific psychological factors related to recovery after surgery. Psychological status was identified from documented psychiatric diagnoses and patient self-report, and recovery was assessed using WORC and ASES scores. Patients presenting with greater distress tended to report worse pain and function before surgery. A recorded history of depression or anxiety was linked to lower ASES and WORC scores both pre- and postoperatively. Despite this, the degree of improvement following surgery was broadly similar to that seen in patients without such a history. When shoulder-specific depression and anxiety were examined using WORC subscales, poorer ASES scores were again observed before and after surgery. However, these patients appeared to experience larger absolute gains over time, likely reflecting recovery from a lower starting level. Correlation coefficients were not reported. The relatively high attrition rate of 37% introduces some uncertainty, and the use of mixed ascertainment methods may have influenced classification.

### 3.3 Anxiety, Depression, and Patient-reported Outcome Measures (PROMs)

In their prospective cohort study, Longo *et al.* [7] assessed preoperative anxiety and depressive symptoms in patients undergoing rotator cuff repair. Psychological distress was measured using the HADS, and outcomes were evaluated with ASES, WORC, and VAS scores. Higher levels of anxiety and depression before surgery were associated with significant postoperative change in both psychological measures and shoulder-specific outcomes. Changes in depressive symptoms showed a moderate correlation with WORC and ASES scores. Taken together, these findings indicate a link between mental health status and functional recovery over time.

### 3.4 Emotional Well-being and Narcotic Use

Ravindra *et al.* [26] evaluated emotional well-being and preoperative narcotic use in relation to longer-term pain outcomes after surgery. Emotional health was measured with the SF-36 Mental Component Score. Patients with poorer emotional well-being and those using opioids preoperatively were more likely to report persistent moderate-to-severe pain at one year. In this cohort, psychosocial variables showed stronger relationships with pain outcomes than structural pathology or intraoperative findings. Correlation coefficients were not provided, but multivariable analysis supported a role for emotional health in the development of chronic pain.

Similarly, Lazaridou *et al.* [27] examined risk factors for chronic postsurgical pain using a biopsychosocial framework. Preoperative negative affect, assessed using the anxiety/depression dimension of the EQ-5D-5L, together with higher baseline pain intensity and poorer shoulder function, was independently associated with persistent pain at six months after surgery. Surgical characteristics were not significantly related to pain persistence. These findings further highlight the relevance of patient-reported psychosocial and symptom-related factors in identifying individuals at risk of adverse pain trajectories.

### 3.5 Psychological Distress and Surgical Impact

In contrast to other findings, Potter *et al.* [15] reported no significant association between mild-to-moderate psychological distress and postoperative outcomes. The study assessed distress using the DRAM and measured postoperative outcomes using the ASES, SST, and VAS for pain. While psychological symptoms tended to improve after surgery, baseline distress was not significantly associated with postoperative pain or function. These findings suggest the possibility of a threshold effect, wherein only more severe psychological burden meaningfully influences recovery outcomes.

### 3.6 Resilience as a Predictive Factor

Wilson *et al.* [28] conducted a prospective cohort study examining psychological resilience in patients undergoing rotator cuff repair, using the BRS. Resilience was not significantly associated with shoulder-specific outcomes, including ASES and QuickDASH scores. A moderate correlation was observed with global mental health recovery measured by the PROMIS Global-10 instrument. Resilience was not associated with localized shoulder function but was related to overall psychological recovery during the postoperative period.

### 3.7 Overall Psychological Profile and Functional Recovery

Thorpe *et al.* [29] assessed overall psychological status using a comprehensive set of measures, including the DASS, PCS, PSEQ, and TSK-11. The primary outcome was the ASES score. Lower baseline psychological scores were linked with reduced function at three, six, and twelve months after surgery. Structural repair findings were similar between groups. However, patients with poorer psychological status described their recovery less favorably. Correlation coefficients were not reported. Stratified analyses still suggested a relationship between psychological status and postoperative outcomes.

Along similar lines, Oba *et al.* [30] evaluated multiple psychosocial constructs, including pain catastrophizing and kinesiophobia, in relation to postoperative functional recovery. Lower preoperative PCS and TSK-11 scores were independently associated with higher postoperative ASES scores. Psychological measures and functional outcomes both improved following surgery [31]. Although the explanatory strength of these models was modest, the findings support the contribution of multidimensional psychological factors to patient-reported recovery.

### 3.8 Summary of Results

Across the studies reviewed, psychological factors before surgery were repeatedly associated with differences in postoperative pain and functional recovery after rotator cuff repair. Where statistical associations were reported, these were generally of small-to-moderate magnitude and may not account for recovery in isolation. Higher levels of depression, anxiety, pain catastrophizing, poorer emotional well-being, and lower resilience were commonly linked with worse postoperative outcomes. However, the pattern was not completely consistent. Some patients with milder distress improved substantially after surgery. In a few cases, broader traits such as resilience appeared to relate more to overall recovery experience than to shoulder-specific outcome scores alone. One study also suggested a possible gene-environment interaction involving COMT genotype and pain catastrophizing. This finding complicates the picture, implying that biological predisposition and psychological state may interact rather than operate independently.

Overall, the evidence suggests that psychological health plays a meaningful role in recovery, but not in a simple or uniform way. It likely shapes how patients experience and report pain and function after surgery. For that reason, considering psychological assessment as part of standard preoperative evaluation may be reasonable, particularly for patients already demonstrating higher levels of distress.

## 4 DISCUSSION

This systematic review synthesizes current evidence on the relationship between preoperative psychological distress and postoperative pain and functional outcomes in patients undergoing rotator cuff repair. Across the ten included non-randomized studies, associations were frequently observed between elevated preoperative psychological distress (particularly depression, anxiety, pain catastrophizing, and poor emotional well-being) and poorer postoperative pain and functional outcomes. These findings support a biopsychosocial model of postoperative recovery and emphasize the role of psychological status as an important factor associated with postoperative outcomes [24].

Psychosocial factors emerged as meaningful associated factors of both acute and persistent postoperative pain and recovery-related outcomes. Simon *et al.* [21] found that higher pain catastrophizing scores were moderately correlated with greater PROMIS pain interference, while depressive symptoms measured with the PHQ-9 were also significantly correlated. Mediation analysis further suggested that depressive symptoms may account for a substantial proportion of the total effect of catastrophizing on 12-month movement-evoked pain. These associations remained significant after adjusting for baseline pain and function, reinforcing the potential prognostic relevance of preoperative psychological distress.

Simon *et al.* [21] also reported a possible gene–environment interaction involving COMT genotype and pain catastrophizing. Their findings raise the possibility that psychological risk factors may interact with biological predispositions in shaping postoperative recovery rather than acting independently. Similar observations have been reported in chronic pain research, where catastrophizing has consistently been associated with increased pain perception and disability [22].

The overall findings across the included studies were not entirely consistent. Although higher psychological distress was often associated with poorer postoperative outcomes, the strength and direction of this relationship varied. Potter *et al.* [15] found that mild-to-moderate distress was not associated with worse ASES, SST, or VAS scores following surgery. Similarly, Cho *et al.* [9] observed that baseline depression and anxiety were not significantly associated with postoperative pain or functional outcomes despite improvements in psychological symptoms after surgery. These findings suggest that the relationship between psychological distress and recovery may not be linear and may depend on severity, chronicity, or the specific psychological construct assessed.

Several studies also indicated that psychological distress decreased following surgical intervention. Lau *et al.* [6] reported that although patients with depression and anxiety had worse absolute postoperative scores, they still experienced meaningful improvement compared with baseline. Longo *et al.* [7] likewise demonstrated that reductions in anxiety and depressive symptoms were moderately correlated with improvements in functional outcomes. Together, these findings suggest that psychological distress does not preclude surgical benefit and that improvements in mental health may occur alongside gains in shoulder function.

Emotional health appears particularly relevant in the transition from acute postoperative pain to persistent pain states. Ravindra *et al.* [26] found that poorer emotional well-being was associated with long-term pain outcomes, while Lazaridou *et al.* [27] further demonstrated that preoperative negative affect and higher baseline pain were independently associated with chronic postsurgical pain. These findings align with broader pain literature indicating that affective vulnerability contributes to pain persistence beyond structural pathology. Collectively, these support the view that recovery trajectories may be influenced not only by surgical factors but also by psychological and symptom-related profiles.

Wilson *et al.* [28] introduced resilience as a potentially protective psychological factor. Although resilience was not associated with shoulder-specific functional outcomes, it correlated with broader measures of global recovery, suggesting that certain psychological traits may influence overall perceptions of health and well-being more than joint-specific outcomes. Thorpe *et al.* [29] extended this perspective by demonstrating that poorer overall psychological profiles across multiple constructs were associated with consistently worse postoperative outcomes. Similarly, Oba *et al.* [30] highlighted the relevance of multidimensional psychosocial constructs such as catastrophizing and kinesiophobia, with lower baseline scores associated with better postoperative functional recovery. These findings suggest that comprehensive psychological assessment may provide greater prognostic insight than isolated mood measures alone.

Beyond pain and functional scores, psychological factors may influence wider recovery domains. Evidence indicates that expectations, participation in rehabilitation, and occupational outcomes are shaped by mental health status. Gowd *et al.* [32] reported that preoperative mental health scores predicted return to work after rotator cuff repair, illustrating that psychological screening has implications extending beyond immediate surgical recovery. Johnson *et al.* [10] similarly observed that preexisting depression and anxiety were associated with poorer postoperative outcomes across cohorts. Moreover, while pre-operative psychological factors may influence post-operative outcomes, as discussed earlier, this relationship may be bidirectional, with chronic pain and disability contributing to psychiatric comorbidity [18].

The findings of this review carry important implications for clinical practice. They suggest that psychological screening may have potential value in selected clinical contexts, and highlight the possibility that targeted psychological interventions may represent an avenue for improving recovery, although direct evidence in surgical populations remains limited. Approaches such as cognitive-behavioural therapy, pain neuroscience education, and mindfulness-based strategies have demonstrated benefits in chronic pain populations and may represent adjunctive strategies within surgical care pathways [33]. Because psychological factors are modifiable, integrating mental health support into perioperative care may offer a low-risk strategy to enhance outcomes, while also acknowledging that these factors may be part of a more complex and dynamic relationship influencing recovery and patient outcomes.

All studies included in this review were observational in design, as no randomised controlled trials met the inclusion criteria. While this reflects the current state of the evidence base in this area, it has important implications for interpretation. Observational studies are inherently limited in their ability to establish causality, and the associations identified between psychological factors and outcomes should therefore be interpreted with caution. These relationships may be influenced by residual confounding, selection bias, and measurement bias. Furthermore, the potential for bidirectional relationships should be considered, whereby psychological factors may both influence and be influenced by the outcomes of interest, making directionality difficult to determine. As a result, the findings of this review are best understood as demonstrating associations rather than causal effects. Nevertheless, observational evidence remains valuable in this context, particularly where experimental designs may be impractical or unethical, and provides an important foundation for hypothesis generation. Future research using longitudinal and, where feasible, interventional designs will be important to further clarify causal pathways.

There are several limitations to consider. As discussed above, all included studies were observational, limiting causal inference. Psychological constructs and outcome measures varied across studies, reducing comparability and precluding formal meta-analysis. Sample sizes differed, and attrition was present in several cohorts, including Lau *et al.* [6]. Risk of bias assessments indicated moderate to serious concerns, particularly regarding confounding and outcome measurement. These factors reduce certainty in the overall conclusions.

This review also has methodological limitations. Three electronic databases (MEDLINE, Embase, and PsycINFO) were searched, and inclusion was restricted to English-language peer-reviewed publications. Relevant studies may therefore still have been missed, particularly unpublished or non-English literature. Additionally, the search strategy may not have captured all relevant studies, particularly those that assessed relevant baseline or psychosocial variables without explicitly using preoperative terminology. This should be considered when interpreting the findings. The number of included studies remained relatively small (n = 10), and heterogeneity in study design and measurement approaches limits generalizability. Heterogeneity precluded the conduction of a meta-analysis. Although broadly similar trends were observed, the magnitude and clinical significance of associations should be interpreted cautiously. In terms of quality assessment tools, we recognize that the Newcastle-Ottawa Scale has limitations when applied to prognostic and clustering studies; however, given the predominance of observational designs in this literature, it was selected as a pragmatic quality assessment tool. Finally, we aimed to evaluate psychosocial factors more broadly. Although psychological variables are increasingly studied in shoulder arthroplasty, socioeconomic and occupational factors such as employment status, social support, education level, and work demands remain underrepresented in the current literature and may contribute to postoperative recovery variability, which was not accounted for in our review.

## CONCLUSION

This review examined whether psychological factors present before surgery are related to outcomes after rotator cuff repair. Across the included studies, there was a general trend suggesting that patients with higher levels of depression, anxiety, pain catastrophizing, negative affect, or broader psychological distress tended to report greater postoperative pain and lower functional scores. The strength of these associations was moderate and not identical across all cohorts, and some studies found no clear predictive relationship. At the same time, psychological distress did not prevent meaningful postoperative improvement, and psychological symptoms often improved alongside functional recovery.

These findings suggest that recovery appears to be influenced by a complex interplay between structural and biopsychosocial factors. Preoperative psychological assessment may therefore help identify patients who could benefit from additional perioperative support, although stronger and more consistent evidence is required before routine changes to clinical practice can be recommended. Future research should further clarify the role of multidimensional psychological constructs and explore whether targeted psychological interventions can modify postoperative pain trajectories and functional recovery. Lastly, future research would benefit from larger population-based designs, including linked registry or cohort studies, which allow for longitudinal assessment of exposures and outcomes over time. Such approaches have been successfully applied in other areas of musculoskeletal research to examine bidirectional relationships between physical and health outcomes at scale [34], and could provide a valuable framework for advancing understanding in this field. In particular, these designs may help disentangle whether psychological factors act as predictors, consequences, or mutually reinforcing components of post-operative outcomes following rotator cuff repair.

## Figures and Tables

**Fig. (1):**
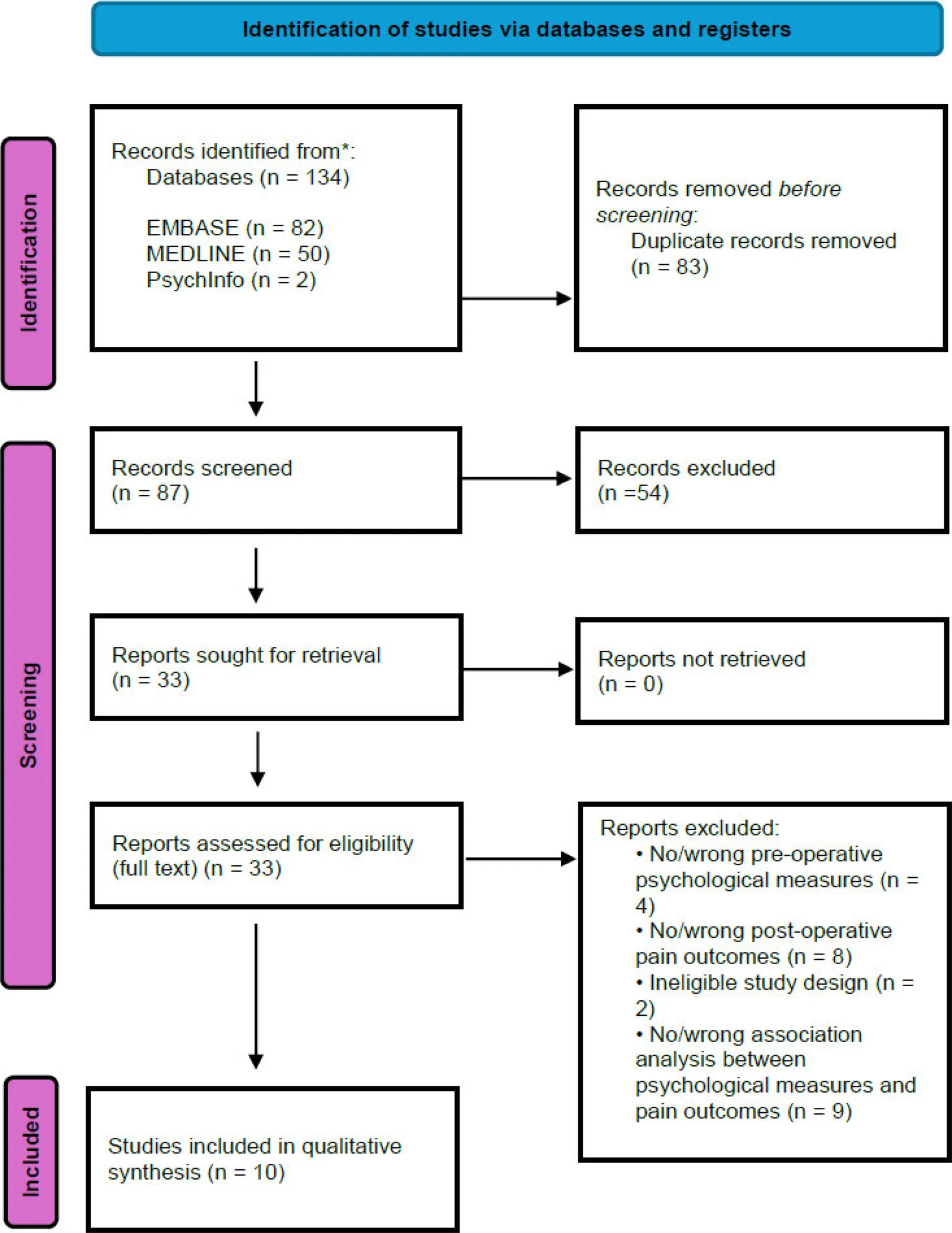
PRISMA flow diagram of study selection.

**Table 1:** Summary of included studies.

**Study (Author)**	**Sample Size**	**Pre-operative Psychological Factor(s) and Tool(s)**	**Post-operative Outcome Measure(s) and Tool(s)**	**Main Findings**	**Association/ Coefficient**	**Strength of Evidence**
Simon *et al.* (2020) [21]	148	Pain Catastrophizing (PCS)	Movement-Evoked Pain (NRS); PROMIS Pain Interference	Higher PCS and COMT genotype associated with worse 12-month pain	Not reported	Moderate to Strong
		Depressive Symptoms (PHQ-9)		Depressive symptoms mediated 53% of the risk effect on 12-month pain	β = 0.53 (*p* = 0.038)	
		Fear of Movement (TSK-11)		Higher TSK scores associated with worse pain at 12 months	R^2^ = 0.1–0.2 (*p* < 0.01)	
Lau *et al.* (2019) [6]	187	Preexisting Depression/Anxiety (clinical diagnosis)	ASES; WORC	Associated with lower pre- and post-op ASES and WORC; distressed patients still improved post-op	*p* = .014, .008 (no coefficient)	Moderate
		Shoulder-specific Depression (WORC)		Negatively correlated with ASES	r = –0.76 (pre-op); r = –0.31 (post-op)	
		Shoulder-specific Anxiety (WORC)		Negatively correlated with ASES	r = –0.732 (pre-op); r = –0.31 (post-op)	
Longo *et al.* (2023) [7]	43	Anxiety/Depression (HADS)	ASES; WORC; VAS	Higher baseline anxiety/depression predicted worse outcomes; reductions in HADS correlated with gains in WORC and ASES	r = 0.48 (WORC), r = 0.44 (ASES), *p* < 0.05	Moderate
Ravindra *et al.* (2018) [26]	93	Emotional Well-being (SF-36 MCS)	VAS	Poorer emotional well-being predicted persistent pain at 1 year; stronger predictors than structural pathology	*p* < 0.05	Moderate
		Pre-op Narcotic Use		Associated with higher 1-year pain	*p* < 0.05	
Potter *et al.* (2015) [15]	85 (70 at 1 yr)	Psychological Distress (DRAM)	VAS; SST; ASES	Mild/moderate distress not associated with outcomes; distress decreased after surgery; no significant differences in pain	*p* = 0.005 (reduction in distress)	Moderate
Wilson *et al.* (2022) [28]	98	Resilience (BRS)	ASES; QuickDASH; PROMIS Global-10	Resilience not correlated with ASES/QuickDASH; positively correlated with PROMIS Global-10	r = 0.35–0.56 (*p* = 0.0025–0.009)	Moderate
Thorpe *et al.* (2018) [29]	124	Depression, Anxiety, Stress (DASS)	ASES	Poorer psych profile predicted worse ASES at all follow-ups (3–12 mo); multidimensional profile stronger predictor than single constructs	Δ ASES: –9 to –15	Moderate
		Pain Catastrophizing (PCS)		Higher PCS linked to worse outcomes	Not reported	
		Self-Efficacy (PSEQ); Fear of movement (TSK-11)		Lower PSEQ, higher TSK linked to worse function	Not reported	
Oba *et al.* (2020) [30]	87	Pain Catastrophizing (PCS)	ASES	Higher catastrophizing predicted poorer function	*p* < 0.05	Moderate
		Self-Efficacy (PSEQ); Fear of movement (TSK-11)		Lower PSEQ, higher TSK linked to worse function	*p* < 0.05	
Lazaridou *et al.* (2023) [27]	90	Negative affect (EQ-5D-5L)	NRS pain	Higher negative affect predicted higher pain	r = 0.26 (*p* < .001)	Moderate
Cho *et al.* (2015) [9]	123	Anxiety and Depression (HADS)	VAS; ASES; UCLA shoulder score; WHOQOL-BREF	Depression and anxiety did not predict postoperative pain or functional outcome	Not reported	Moderate

**Note:** **Pre-operative psychological factors/tools:** BRS = Brief Resilience Scale; COMT = Catechol-O-methyltransferase; DASS = Depression Anxiety Stress Scale; DRAM = Distress and Risk Assessment Method; HADS = Hospital Anxiety and Depression Scale; PCS = Pain Catastrophizing Scale; PHQ-9 = Patient Health Questionnaire-9; PSEQ = Pain Self-Efficacy Questionnaire; SF-36 MCS = Short Form-36 Mental Component Score; TSK-11 = Tampa Scale of Kinesiophobia (11-item version); WORC = Western Ontario Rotator Cuff Index. **Post-operative outcome measures/tools:** ASES = American Shoulder and Elbow Surgeons score; NRS = Numeric Rating Scale; PROMIS = Patient-Reported Outcomes Measurement Information System; PROMIS Global-10 = PROMIS Global Health Short Form (10 items); QuickDASH = Quick Disabilities of the Arm, Shoulder and Hand; SST = Simple Shoulder Test; UCLA = University of California Los Angeles Shoulder Score; VAS = Visual Analog Scale; WHOQOL-BREF = World Health Organization Quality of Life Scale - Brief Version.

**Table 2:** ROBINS-I risk of bias assessment for included studies.

**Study**	**Confounding**	**Selection**	**Classification**	**Deviations**	**Missing Data**	**Measurement**	**Reporting**	**Overall Risk**
Simon *et al.* (2020) [21]	Low	Low	Low	Low	Low	Low	Low	Low
Lau *et al.* (2019) [6]	Moderate	Serious	Moderate	Low	Serious	Low	Low	Moderate–Serious
Longo *et al.* (2023) [7]	Moderate	Low	Low	Low	Low	Low	Low	Moderate
Ravindra *et al.* (2018) [26]	Serious	Moderate	Moderate	Low	Low	Low	Low	Moderate–Serious
Potter *et al.* (2015) [15]	Moderate	Low	Low	Low	Low	Low	Low	Moderate
Thorpe *et al.* (2018) [29]	Moderate	Low	Low	Low	Low	Low	Low	Moderate
Wilson *et al.* (2022) [28]	Moderate	Low	Low	Low	Low	Low	Low	Moderate
Cho *et al.* (2015) [9]	Moderate	Moderate	Low	Low	Moderate	Low	Low	Moderate
Oba *et al.* (2020) [30]	Moderate	Moderate	Low	Low	Low	Low	Low	Moderate
Lazaridou *et al.* (2023) [27]	Moderate	Low	Low	Low	Moderate	Low	Low	Moderate

**Table 3:** Quality assessment of included studies using the newcastle-ottawa scale (NOS).

**Study**	**Selection (0–4)**	**Comparability (0–2)**	**Outcome (0–3)**	**Total (0–9)**	**Quality Grade**
Simon *et al.* (2020) [21]	4	2	3	9	High
Lau *et al.* (2019) [6]	3	1	2	6	Moderate
Longo *et al.* (2023) [7]	4	1	3	8	High
Ravindra *et al.* (2018) [26]	3	1	2	6	Moderate
Potter *et al.* (2015) [15]	4	1	3	8	High
Thorpe *et al.* (2018) [29]	4	1	3	8	High
Wilson *et al.* (2022) [28]	4	1	3	8	High
Cho *et al.* (2015) [9]	3	1	2	6	Moderate
Oba *et al.* (2020) [30]	4	1	3	8	High
Lazaridou *et al.* (2023) [27]	4	1	3	8	High

## Data Availability

All the data and supportive information is provided within the article.

## LIST OF ABBREVIATIONS

ASES: American Shoulder and Elbow Surgeons score
BRS: Brief Resilience Scale
COMT: Catechol-O-methyltransferase
DASS: Depression Anxiety Stress Scale
DRAM: Distress and Risk Assessment Method
EQ-5D-5L: EuroQol 5-Dimensions 5-Levels questionnaire
HADS: Hospital Anxiety and Depression Scale
NOS: Newcastle-Ottawa Scale
NRS: Numeric Rating Scale
NRT: Non-Randomized Trial
PCS: Pain Catastrophizing Scale
PHQ-9: Patient Health Questionnaire-9
PRISMA: Preferred Reporting Items for Systematic Reviews and Meta-Analyses
PROMS: Patient-Reported Outcome Measures
PROMIS: Patient-Reported Outcomes Measurement Information System
PROMIS Global-10: Patient-Reported Outcomes Measurement Information System Global Health Short Form (10 items)
PROSPERO: International Prospective Register of Systematic Reviews
PSEQ: Pain Self-Efficacy Questionnaire
QuickDASH: Quick Disabilities of the Arm, Shoulder and Hand
RCT: Randomized Controlled Trial
ROBINS-I: Risk of Bias in Non-randomized Studies of Interventions
SF-12: 12-Item Short Form Health Survey
SF-36: 36-Item Short Form Survey
SF-36 MCS: 36-Item Short Form Survey Mental Component Score
SPADI: Shoulder Pain and Disability Index
SST: Simple Shoulder Test
TSK-11: Tampa Scale of Kinesiophobia-11
UCLA: University of California Los Angeles shoulder score
VAS: Visual Analog Scale
WHOQOL-BREF: World Health Organization Quality of Life Scale – Brief Version
WORC: Western Ontario Rotator Cuff Index

## STANDARDS OF REPORTING

PRISMA guidelines and methodology were followed.

## SUPPLEMENTARY MATERIAL

PRISMA checklist is available as supplementary material on the publisher’s website along with the published article.

## APPENDIX 1

Appendix 1: Database-specific search strategies.

**MEDLINE (*via* OVID)** Search date: 6^th^ March 2026 Search string: (("rotator cuff surgery" or "rotator cuff repair") and pain and preoperative and (psychological or depression or "pain catastrophizing" or anxiety or kinesiophobia or "psychological factors" or emotional or "mental health")).ti,ab.

**Embase (*via* OVID)** Search date: 6^th^ March 2026 Search string: (("rotator cuff surgery" or "rotator cuff repair") and pain and preoperative and (psychological or depression or "pain catastrophizing" or anxiety or kinesiophobia or "psychological factors" or emotional or "mental health")).ti,ab.

**PsycINFO** Search date: 6^th^ March 2026 Search string: (("rotator cuff surgery" or "rotator cuff repair") and pain and preoperative and (psychological or depression or "pain catastrophizing" or anxiety or kinesiophobia or "psychological factors" or emotional or "mental health")).ti,ab.
